# Effects of Advertising on Food Consumption Preferences in Children

**DOI:** 10.3390/nu12113337

**Published:** 2020-10-30

**Authors:** José Antonio Ponce-Blandón, Manuel Pabón-Carrasco, Rocío Romero-Castillo, Macarena Romero-Martín, Nerea Jiménez-Picón, María de las Mercedes Lomas-Campos

**Affiliations:** 1 Red Cross Nursing School, University of Seville, 41009 Seville, Spain; japonce@cruzroja.es (J.A.P.-B.); mpabon@cruzroja.es (M.P.-C.); mromero@cruzroja.es (M.R.-M.); nejipi@cruzroja.es (N.J.-P.); 2 Faculty of Nursing, Physiotherapy and Podiatry, University of Seville, 41009 Seville, Spain; mlomas@us.es

**Keywords:** advertising, food publicity, food preferences, fast foods, child behavior

## Abstract

(1) Background: Childhood obesity is a public health problem. The purpose of this study was to know if exposure to commercial messages which advertise food products exerts any effect on the short-term consumption preferences of 4- to 6-year-old children. (2) Methods: A double-blind and randomized experimental design. Sample consisted of 421 boys and girls from twelve schools in a city in Spain. (3) Results: In three of the four product pairs shown, the products advertised in the intervention were preferred. In the results of applying the model for the first product pair presented, sugared cereals, the predictive variable which best explains the behavior of the preferences expressed is gender (Odds Ratio 0.285 (0.19–0.42); *p* < 0.05). For the second pair, chocolate cookies, the family’s nationality has a strong weight in the model. As regards the regression model calculated for the last pair (filled rolls), the predictive variable which showed having more influence was gender. Boys had a 1.39 times higher risk of selecting the advertised product than girls. (4) Conclusions: The persuasive effect of commercials has shown to be influential in a general, immediate, and significant way only in the case of products with wide brand awareness. This study reinforces the importance of advertising and emphasizes the need to initiate measures to control the content of TV commercials.

## 1. Introduction

Childhood obesity is a public health problem. Overweight five-year-old children have a four to five times higher risk of being overweight in adolescence, and a significantly higher risk of obesity in adulthood [1]. According to the World Health Organization (WHO), the prevalence of obesity has risen at an alarming pace; it is estimated that, in 2016, more than 41 million children under the age of five worldwide were overweight or obese [2]. This population has more probabilities of suffering non-transmissible diseases at early ages, like diabetes and cardiovascular diseases. If the current trends are maintained, in 2022, there will be more obese children and adolescents than those with weight deficit and, in 2025, the world prevalence of obesity will reach 18% in men and will exceed 21% in women [3]. In Spain, the prevalence of childhood obesity is 34%, fourth place in the European Union countries [4]. The health bodies have requested regulatory measures which restrict the marketing of harmful foods for the health of children, including the advertising of unhealthy food products on television [2,5,6].

Zimmerman and Bell (2010) concluded that watching TV commercials was related to a higher Body Mass Index, whereas watching television without commercials was associated with a lower score in the Body Mass Index [7]. Simultaneously, fast food advertising has also been associated with the increase in the intake of unhealthy food products and in Body Mass Index [8]. Fast food, characterized by a large content of saturated fats and sugars, contributes to overweight and to incorrect nutrition in children [9]. Therefore, reducing the intake of fast food during preschool and school years is a simple way to improve children’s health.

The companies which produce fast food target children through attractive tactics, including bonds to renowned and admired characters and offering gifts when buying the products [10]. Additionally, it has been verified that the commercials in which a person appears consuming the product have more influence on the perception of consumers than those in which only the product is presented [11]. One of the effects of this type of commercial in children is imitation and the possibility of imagining and wishing to consume the product themselves [12,13,14]. Television is the main communication media used by companies to promote unhealthy food products [15], aimed at a vulnerable audience incapable of understanding the persuasive intention of advertising [16].

Studies on the effect on behavior have investigated the degree to which children are persuaded to buy or to ask for the advertised products or brands [17]. Different experimental studies have shown that children are more prone to select branded fast food products in detriment to the unbranded ones, and that they qualify branded food products as tastier than their unbranded equivalents [18,19]. Product advertising seeks to produce cognitive, affective, and/or behavioral responses in the audience, children in this case [20]. Hudson and Elliot (2013) found that the positioning of healthy and unhealthy food products had a slight influence on the immediate behavior [21]. However, van Reijmersdal et al. (2010) demonstrated a positive relation between exposure to brand positioning and the behavior of choosing it [22]. The preference for branded articles has been associated with the higher number of TV sets in the home, which suggests that exposure to TV commercials influences children’s preferences [1,23].

Despite the importance of the effect of TV food commercials on eating habits and on health, there is still a scarce number of studies which directly link food advertising to the intake of food products or to obesity, due to the difficulty in measuring the exposure to the commercials [8]. The studies conducted on this line have shown a weak support for the immediate effect on behavior, measured as willingness to buy or to consume immediately after exposure [20]. The fact that few studies have been conducted in Spain in this field reinforces the need to launch research studies which analyze the impact of advertising on the food consumption preferences of children in Spain. Concretely, the objective of this study is to know if exposure to commercial messages which advertise food products exerts any effect on the short-term consumption preferences of 4- to 6-year-old boys and girls. The main study hypothesis sets out that the children who watch child TV shows with food product commercials express more preferences for the advertised products than those who watch these same child TV shows without any advertising content. Secondarily, diverse hypotheses will be explored which set out possible relations among sex, age, nationality, and consumption preferences.

## 2. Materials and Methods

### 2.1. Study Design and Sampling

This study presents a double-blind and randomized experimental design. The study universe was a population of children between 4- and 6-years-old living in the city of Seville (Spain). This population is enrolled in 172 school centers, according to the statistics from the Education Council of the Andalusia Board [24].

To determine the sample size, the procedure established for hypothesis contrast studies, in which proportions are compared, was used. Specifically, considering that the minimum safety of the study must be 95% (*p* < 0.05), that the test power (1-β) must also be of at least 95%, and using as value references the proportions of food products preferred by the children included in the study by Borzekowski and Robinson (2001) [25], that is, 54% of the children in the intervention group and 42% in the control group, the minimum size of each group had to be of at least 65 children. Taking into account that the choice was to recruit two control groups and two intervention groups, a minimum sample size of 260 children was needed, coming from a total study population of 13,945. To avoid selection bias which could suppose the loss of study subjects due to errors in data collection or to withdrawals, it was proposed to add a 10% safety margin to the sample, eventually leading to a minimum sample size of 286 subjects.

**Figure 1:**
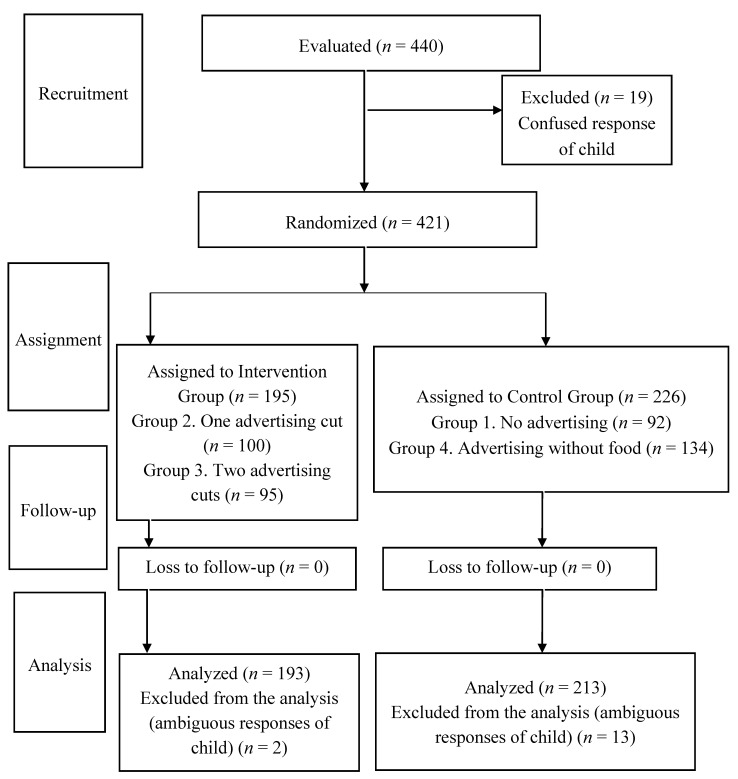
CONSORT flow diagram.

The sampling procedure employed was that of cluster sampling, stratified by center topology (public or private, including those of a concerted character in the latter), with Seville’s Child Education centers constituting the primary sampling units. Using as reference the figure of the mean number of pupils per group or class ratio, which is 23.6 pupils, and assuming that in each center, only one group was studied, it was estimated that a total of 12 centers were needed to complete the sample size, that is, 3 centers for each of the 4 study groups. A flow diagram of the sample recruitment is represented in Figure 1, following the CONSORT model.

The cluster selection of these 12 centers was at random by using the systematic sample technique in each of the subgroups, employing a unit selection interval of 14, since the ratio between the total of centers and the number of centers (172/12) is 14.3. The 12 centers were obtained after selecting a random number obtained in the Microsoft^®^ Excel 2010 application and after applying the 14-unit interval. Subjects who did not have a television at home and children with dietary restrictions and allergic or intolerant to some foods were excluded of the study.

### 2.2. Procedure

The study subjects were randomly allocated to four different groups. The four groups were exposed to watching an 8-min episode of the “Caillou” cartoon series, an animation character which is popular among children. The episode was called “*Caillou bombero*” (“Fireman Caillou”) and was selected with the aim that no reference was made to food consumption in its content. The control groups were groups 1 and 4, so that group 1 was exposed to the same cartoon episode but without commercials, whereas group 4 watched the same episode of “Caillou” with one commercial cut advertising different non-food articles (toys, objects, etc.). Children watched their assigned advertisement as a group on a single TV. Then, they went to a room individually where investigators exposed pairs of products.

The interventions groups were groups 2 and 3, so that group 2 watched this episode with one commercial cut which included 4 commercials of different food products aimed at a child audience. The products selected for advertising insertion were snacks and breakfast preparations of limited nutritional value and high fat and sugar contents. Group 3 watched the same cartoon episode but with two commercial cuts, so that in the second cut, the same four commercials were repeated.

The commercials used for the intervention were selected from those studied in *Análisis de contenido de la publicidad de productos alimenticios dirigida a niños y a adultos en Andalucía* (Content analysis of food product advertising aimed at children and adults) [26,27]. The content of 91 different food product commercials was studied, of which 39 were food commercials aimed at a child-youth audience; 41% advertised cereals, cookies, or cocoa, while 23.1%—dairy products. Two experts from the Andalusia Care Plan for Childhood Obesity were consulted to select the commercials meeting the following criteria [28]:

✓ Commercials of high fat and/or sugar content food products aimed at a child audience.
✓ Products aimed to be consumed at breakfast and included in the most frequently announced food groups: sugared cereals, cookies, cocoa, and dairy products.
✓ Including some commercial which employs special effects or fantastic situations, another which promotes giving some gift, collectible, or present, and a third one which uses the testimony of some famous character.

**Figure 2:**
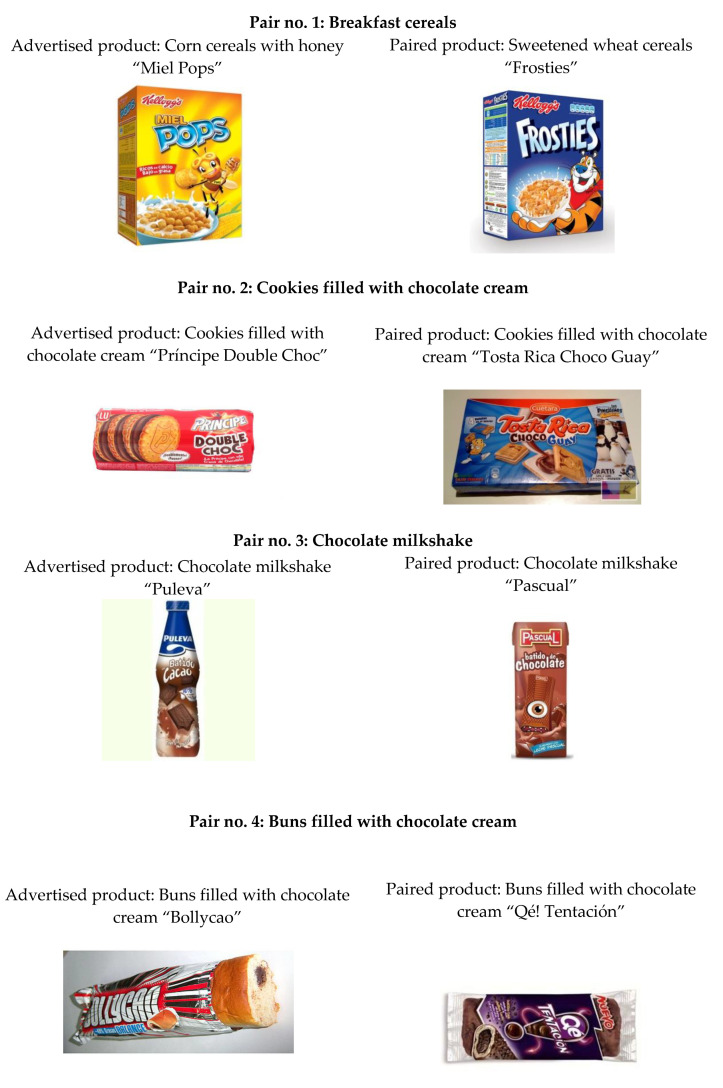
Images of the products used for the choice method.

Thus, the following products were selected (Figure 2):

1. Third commercial: “Miel Pops” cereals.
  Final voiceover with a slogan: “Mielpops, el desayuno más pop” (“Mielpops, the most pop breakfast”). Spot based on the use of shocking images and on fantastic situations by means of animation techniques.
2. Fourth commercial: “Príncipe Double Choc” chocolate cookies.
  Spot in which a famous Spanish soccer player explains the benefit of the product or his experiences related with it: “I like them with a lot of chocolate cream, like my Príncipe Double Choc”. This is a testimonial spot which uses the words of a celebrity as an advertising resource.
3. First commercial: “*Puleva^®^*” cocoa shakes.
  Final voiceover saying: “*Descubre el Club Batidos Puleva y podrás ganar Play Stations, juegos Sing Star y miles de regalos*” (“Discover the Puleva Shake Club and you can win Play Stations, Sing Star games, and thousands of presents”). Advertising version: “*Estos batidos son algo especial: Son batidos Puleva*” (“These shakes are something special. They are Puleva shakes”). Spot based on music and on visual experimentation.
4. Second commercial: “Bollycao” chocolate-filled roll.
  Final voiceover saying: “*Ahora con los Bollycao únete a la banda de ‘Los Simpsons’ con los ‘bollytransfer’*” (“Now with the Bollycaos, join the ‘The Simpsons’ gang with the ‘bollytransfers’”). Spot based on music and on visual experimentation. It also presents a gift inside the wrapping, a “bollytransfer”, which is a sticker with a character from “The Simpsons”.

On the date scheduled, the researcher, together with a surveyor trained on the outcome measurement procedure but unaware of the allocated research group, approached the class tutor.

The children were invited to sit down in a room fitted out to watch cartoons. Afterwards, each child was individually directed to the research area. They were asked to select among some photographs shown in pairs, one of them with the article included in the commercial spot and the other showing a similar product. The products which were paired to the intervention participants were the following:

Product pair No. 1: Breakfast cereals:

Paired product: “Frostis” (*Kellogg’s^®^*) breakfast cereals made with sugared wheat. Advertised product: “Miel Pops” (*Kellogg’s^®^*) breakfast cereals made with popcorn and honey.

Product pair No. 2: Chocolate cream filled cookies:

Paired product: “Tosta Rica Chocoguay” (*Cuétara^®^*) chocolate cream filled cookies. Advertised product: “Príncipe Double Choc” (*Galletas Lu^®^*) chocolate cream filled cookies.

Product pair No. 3: Chocolate shakes:

Paired product: “*Pascual^®^*” chocolate shakes. Advertised product: “*Puleva^®^*” chocolate shakes.

Product pair No. 4: Filled roll:

Paired product: “Qé Tentación” (*Panrico^®^*) chocolate filled roll. Advertised product: “Bollycao” (*Panrico^®^*) chocolate filled roll.

The researcher, who was present at the time of the survey, recorded the child’s answer to each of the four advertised products in a collection sheet. In case the child did not answer or if contradictions were perceived in the answer, it was recorded as “not valid” and the participant was excluded from the study.

### 2.3. Data Collection and Analysis

The following were collected as descriptive general variables of the population’s sociodemographic characteristics: sex, type of center, and nationality; these were analyzed as independent variables. The dependent variables were established by studying the consumption preferences stated by the participants when shown the photographs of the four pairs of food products. The collected data were poured into a questionnaire elaborated with the Epi Info version 7.1.3.10 tool.

For the statistical analysis, the first steps were debugging the database and performing a descriptive analysis. For the frequencies of the qualitative variables, 95% confidence intervals for proportions were calculated, whereas the quantitative variables were numerically summarized by calculating the main measures of central tendency and dispersion. The Kolmogorov–Smirnov test was used to verify the normality of the “summation of the scores” variable based on the preferences from the pairs expressed by the participants. For the hypothesis contrast, Pearson’s χ^2^ test was used, as well as Fisher’s Exact test as necessary, with a *p* value below 0.05 as the significance level. The Risk Ratio (RR) values were calculated, together with their confidence interval for 95%.

Finally, a multivariate analysis was performed to contrast all the predictive variables, which were contemplated in the different bivariate analyses, with assessment of the outcome: the preferences expressed by the participants for each pair of products presented. The multiple logistic regression technique was used to this end. For this, it was necessary to recode the preferences of each product pair to “dummy” variables, in which a value of “0” indicated choosing the non-advertised paired product and a value of “1” indicated choosing the advertised product. The independent variables included pertained to a control or intervention group, sex, and Spanish or foreign nationality. To build the multiple logistic regression model, Odds Ratios were calculated for each of the predictive variables introduced in the model, their confidence intervals for 95%, and the logistic regression coefficient. As well as the result of the statistical test and its significance level, the Newton–Raphson maximum likelihood method was used as a criterion to estimate the parameters. The Epi Info version 7.1.3.1 tool was used for data analysis.

### 2.4. Ethical Considerations

Authorization was obtained from the Research Ethics Committee of the University of Seville, as well as favorable reports from the Integral Care Plan for Childhood Obesity of the Andalusia Board and from the Seville Branch of the Education Council. Information meetings were conducted with parents and educators, and the father’s/mother’s or legal guardian’s informed consent was obtained for each of the study participants. Additionally, anonymity and the protection of personal data were guaranteed to the participants during data collection and analysis.

## 3. Results

In the initial selection, 6 children were excluded for not having television at home and 28 children for having an allergy, intolerance, or dietary restriction. The sample eventually consisted of 421 boys and girls from twelve schools in the city of Seville (Figure 1), with a mean age of 4.8 years (SD = 0.57; (4.74–4.85)). A total of 52% (47.1–56.9) were girls and 48% (43.1–52.9) were boys. Regarding their nationality, 93.1% (90.1–95.2) were Spanish, and the rest came from other countries. A total of 226 subjects participated in the control groups (53.7% (48.8–58.5)), whereas 195 participated in the intervention groups (46.3% (41.5–51.2)).

In relation to the results of the consumption preferences, in three of the four product pairs shown, the products advertised in the intervention were preferred, as can be seen in Table 1. The bivariate contrast of the main hypothesis conducted with the preferences expressed for product pair no. 4 found significant differences between the control and intervention groups (*p* < 0.05). No significant differences were observed for the rest of the products (Table 1).

As regards the hypothesis contrast between sex and consumption preferences, significant differences were observed for two of the product pairs presented. The girls said they mostly preferred the product advertised in pair no. 1, sugared cereals (*p* < 0.05); the boys mostly preferred the product advertised in pair no. 2, chocolate cookies (*p* < 0.05), with a relative risk of 1.98 and 1.54, respectively. As regards age (grouped into children younger and older than 5-years-old), no differences were appreciated in consumption preferences. In relation to nationality (grouped into Spanish and others), the preferences expressed for pair no. 2, chocolate cookies (*p* < 0.05), and pair no. 4, filled rolls (*p* < 0.05), were significant. Children coming from Spanish families preferred the advertised products more frequently than those of other nationalities (Table 2).

A mean score was obtained after recoding the children’s preferences. It was necessary to recode the preferences of each product pair to “dummy” variables, in which a value of “0” indicated choosing the non-advertised paired product, and a value of “1” indicated choosing the advertised product. After coding, the researchers performed a summation of each child’s preferences, obtaining a unique value. In the first comparison, between the control and intervention groups, a mean score of 2.10 (SD = 1.02; (2.00–2.19)) was obtained in the children allocated to the control group, and of 2.17 (SD = 0.98; (2.07–2.26)) in those allocated to the intervention group. The variance values were 1.045 and 0.965, respectively. Once the normality of the outcome variable was verified, the Student’s t parametric test was applied, obtaining a result of 0.7008 which, for the 408 degrees of freedom (the valid answers in this test were 410), granted a significance level of *p* > 0.05. In this way, it was verified that there were no statistically significant differences in the scores obtained by the two groups.

In the comparison of the means and variances of the scores obtained by each of the four allocation groups, it was found that the mean score for group 1 (control without commercials) was 2.076 (SD = 0.97; (1.87–2.27), for group 2 (intervention with one commercial cut) 2.14 (SD = 0.94; (1.95–2.32)), for group 3 (intervention with two commercial cuts) 2.204 (SD = 1.02; (1.99–2.41)), and for group 4 (control with commercials of non-food products) 2.120 (SD = 1.06; (1.93–2.30)). Once the homogeneity of the variances was verified, the ANOVA parametric test was applied. This test showed that there were no statistically significant differences among the scores obtained in the four groups, since the value obtained was 0.262, which, for 3 degrees of freedom, corresponds to a significance level of *p* > 0.05.

In the results of applying the model for the first product pair presented, sugared cereals, the predictive variable which best explains the behavior of the preferences expressed is sex, with an Odds Ratio of 0.285 (0.19–0.42); *p* < 0.05. With equal behavior in the rest of the variables, the girls chose the advertised product 1/0.285 = 3.5 times more frequently (Table 3).

For the second pair, chocolate cookies, the family’s nationality has a strong weight in the model, an Odds Ratio of 0.383 (0.16–0.90) having been obtained, with a significance level of *p* < 0.05. Therefore, boys and girls of Spanish nationality present a 1/0.383 = 2.6 times higher risk of selecting the advertised product, “Príncipe Double Choc” chocolate cookies in this case. Sex once again appears as an important predictive variable in the model (Odds Ratio: 0.457 (0.30–0.68), *p* < 0.05). Consequently, the girls have a 1/0.457 = 2.18 times higher risk of selecting the advertised product than the boys, with equal behavior in the rest of the variables (Table 3). The best multivariate logistic model establishes that: *Logit*(*p*) = 1.162 + (0.003 Group) − (0.959 Nationality) − (0.78 Sex).

In the regression model calculated for the third of the product pairs (cocoa shakes), none of the predictive variables showed any statistically significant influence. The variable which obtained a more influential result on the effect was age, with an Odds Ratio of 1.177 (0.8–1.7), but without a significant level of *p* > 0.05 (Table 3). These results allow choosing the following as the best multivariate logistic model: *Logit*(*p*) = −0.117 − (0.05 Group) + (0.242 Nationality) − (0.385 Sex).

As regards the regression model calculated for the last pair (filled rolls), the predictive variable which showed having more influence on the preferences expressed was sex. The boys had a 1.39 times higher risk of selecting the advertised product than the girls, given equal values in the rest of the variables. Consequently, the Odds Ratio obtained for this variable was 1.299 (0.94–2.07), with a non-significant level (*p* > 0.05) (Table 3). The results shown establish the following as the best multivariate logistic model: *Logit*(*p*) = 0.589 + (0.299 Group) − (0.667 Nationality) + (0.336 Sex).

## 4. Discussion

The aim of this study was to make contributions to the hypothesis which asserts that exposure to advertising messages for food products can have a direct influence on preschool boys’ and girls’ preferences and, indirectly, on their eating habits, from the search of possible risk factors that exert their influence on the high figures of childhood obesity and overweight that have been registered in the last years in our setting.

Various international multi-centric studies with heterogeneous populations have employed experimental methodologies similar to those in this study [29,30,31,32]. The most frequent experimental method is the exposure of the study subjects, in this case the children, to watching a cartoon TV show with commercial cuts in fitted-out rooms in the same school setting. It is a very usual methodology and of proved efficacy to evaluate the effect of advertising on the consumption preferences of children. In this study, the choice was to incorporate two control groups in order to minimize confusion biases. The decision to include a control group whose participants watch the same animation video with commercial cuts which include commercials for other non-food products has been made on account of previous clinical trials with similar hypotheses [33,34]. As regards the outcome assessment, based on the use of pairs of similar products shown to the study subjects, it has also been successfully employed in previous studies [18,31].

With respect to the results obtained, it is verified that, in the 4–6-year-old range, advertising does not seem to have a determinant effect on the immediate preference expressed, save for one of the products presented: the “Bollycao” roll filled with chocolate cream. For this product, advertising was able to exert its influence on the children’s preferences. Finding the immediate relation between advertising and preferences has been a persistent fact in the different stratifications and multivariate analyses performed. In the “Bollycao” commercial, a gift was offered when buying the product, which managed to exert some influence on the children’s choices. Authors like Kraak and Story (2015) have already found a relation between consumption preferences and offering gifts when buying products [10]. In that way, Kelly et al. (2008) particularly corroborated this hypothesis when the products advertised were not essential food products [35]. In this case, the brand’s memorability has also imposed some specific weight, since it manages to reach a phenomenon which Advertising calls “exclusivity of the brand” [36]. This means that a large part of Spanish society calls all industrial chocolate-filled rolls “Bollycao”.

The choice frequencies of the advertised product in the experimental group exceeded 50% for three of the products (“Bollycao”, “Miel pops” sugared cereals, and “Puleva” cocoa shakes), which denotes the general knowledge of these products by the participating children. In the United Kingdom, Boyland et al. (2011) verified that the children preferred food products with high fat or sugar contents if they were of a brand recognized from TV commercials [37]. Longacre et al. (2017) verified that exposure to TV commercials of cereals for children is associated with the family buying the advertised product [38]. According to Castetbon et al. (2012), households are 13 times more likely to buy those cereals aimed at children advertised on TV than unbranded cereals [39].

As regards the second product pair, chocolate cookies, the Spanish nationality presents a high weight on the regression model calculated. Boys and girls of Spanish nationality present a 2.6 times higher risk of selecting the advertised product, “Príncipe Double Choc cookies” in this case. This can be due to the presence of a renowned Spanish soccer player in the commercial. As already confirmed by Poor et al. (2013), commercials in which a person appears consuming the product have more influence on the perception of consumers than those in which only the product is presented [11]. This influence increases when the product is consumed by famous people, like sports celebrities [40].

The sex differences are consistent even when the sample is stratified by control and intervention groups. Many manufacturers from the food industry design products focused specifically on female and male child audiences. This has also been previously contrasted by Sixsmith and Furnham (2010), who found that, in the healthiest food commercials aimed at the child audience, more female characters and voices were used to attribute healthy properties to more feminine wishes [41], whereas those aimed at boys made use of sports celebrities [40]. This is a fact which has been contrasted in our study with the chocolate cookies product pair. Other authors concluded that children were more likely to choose foods with gender-consistent packaging and hypothesized that this effect may be explained by social norms. Children are motivated to fit into the gender expectations that they perceive [42].

### Limitations

One of the limitations of this study was assigning the preference expressed by the child, due to the age characteristics of the population. To this end, interviewers with experience in these age groups were selected. During the data collection period, some cases of uncertain answers were recorded, in concrete from 19 children, who were discarded from the sample to guarantee maximum debugging and quality of the results. Nevertheless, the consistency of the results could have been improved by assessing the outcome at two different times, with a pre-test and a post-test. However, this way to proceed is justified by the difficulty in making correct assessments in the young age range of the study population.

On the other hand, the influence of advertising demonstrated in this study is not as conclusive as in previous studies, as it has only been partially contrasted. This can be due to the fact that advertising is not the only factor related to eating habits and to consumption preferences but also family, educational, socioeconomic, and personal factors [41,43]. Because children are so heavily exposed to junk food advertising in the course of their normal lives, it can be challenging to detect the effects of brief experimental advertising interventions.

## 5. Conclusions

The persuasive effect of the commercials on the 4- to 6-year-old children under study has shown to be influential in a general, immediate, and significant way only in the case of products with wide brand awareness and a historical selling experience in the market.

This study reinforces the importance of advertising to a very vulnerable collective and emphasizes the need to initiate measures to control the deceitful and disturbing content of TV commercials. In this sense, further experimental research studies are needed to detect influences in order to better control and regulate the advertising of food products to the child audience, especially when the products’ nutritional values are not desirable.

## Figures and Tables

**Table 1:** Distribution of consumption preferences for the sample as a whole and bivariate analyses by group.

	Product	Total (N = 421) n (%)	Control Group (n = 200) n (%)	Intervention Group (n = 191) n (%)	RR (CI 95%)	*p*
Pair No. 1 Sugary cereals	Advertised: “Miel pops”	231 (55.3)	124 (55.6)	107 (54.9)	0.98 (0.79–1.21)	0.8803
	Not advertised: “Frostis Kellogg’s”	187 (44.7)	99 (44.4)	88 (45.1)		
Pair No. 2 Chocolate cookies	Advertised: “Príncipe Double Choc”	184 (43.7)	100 (44.2)	84 (43.1)	1.02 (0.82–1.27)	0.8091
	Not advertised: “Tosta Rica Choco Guay”	237 (56.3)	126 (55.8)	111 (56.9)		
Pair No. 3 Chocolate milkshake	Advertised: “Batidos Puleva”	270 (64.9)	147 (65.9)	123 (63.7)	1.03 (0.89–1.19)	0.6408
	Not advertised: “Batidos Pascual”	146 (35.1)	76 (34.1)	70 (36.3)		
Pair No. 4 Buns filled with chocolate	Advertised: “Bollycao”	212 (51.2)	102 (46.8)	106 (56.2)	0.82 (0.68–0.99)	0.0448
	Not advertised: “Qé Tentación”	202 (48.8)	117 (53.2)	89 (43.8)		

**Table 2:** Bivariate analyses of consumption preferences by sex and nationality.

Variables	Categories	PAIR No. 1 Sugary Cereals		PAIR No. 2 Chocolate Cookies		PAIR No. 3 Chocolate Milkshake		PAIR No. 4 Buns Filled with Chocolate	
		“Miel pops”	Frosties Kellogg’s	Príncipe Double Choc	Tosta Rica Choco Guay	Milkshake Puleva	Milkshake Pascual	Bollycao	Qé Tentación
Sex	Boys	80 (39.8%)	121 (60.2%)	133 (65.8%)	69 (34.2%)	121 (60.5%)	79 (39.5%)	111 (55.5%)	89 (44.5%)
	Girls	151 (69.6%)	66 (30.4%)	104 (47.5%)	115 (52.5%)	149 (69.0)	67 (31.0%)	101 (47.2%)	113 (52.8%)
	RR (CI 95%) *p*	1.98 (1.57–2.49) 0.00000		1.54 (1.22–1.93) 0.00015		1.14 (0.98–1.31) 0.0701		0.85 (0.70–1.02) 0.0912	
Nationality	Spanish	214 (55.2%)	174 (44.8%)	176 (45.0%)	215 (55.0%)	249 (64.5%)	137 (35.5%)	202 (52.6%)	182 (47.4%)
	Other nationalities	16 (55.2%)	13 (44.8%)	8 (27.6%)	21 (72.4%)	20 (69.0%)	9 (31.0%)	10 (34.5%)	19 (65.5%)
	RR (CI 95%) *p*	1.03 (0.67–1.58) 0.8725		1.68 (0.92–3.08) 0.0499		0.92 (0.72–1.17) 0.5437		1.57 (0.94–2.64) 0.0419	

**Table 3:** Multiple logistic regression model for consumption preferences.

Predictor Variable	Odds Ratio	CI 95%	Coef.	Standard Error	Statistical	*p*
Pair No. 1						
Group (intervention/control)	1.084	(0.69–1.69)	0.080	0.22	0.355	0.722
Nationality	0.978	(0.43–2.17)	−0.021	0.40	−0.052	0.958
Sex	0.285	(0.19–0.42)	−1.252	0.20	−6.015	0.000
Pair No. 2						
Group (intervention/control)	1.003	(0.64–1.52)	0.003	0.222	0.017	0.986
Nationality	0.383	(0.27–0.94)	−0.959	0.440	−2.176	0.0295
Sex	0.457	(0.30–0.68)	−0.781	0.204	−3.822	0.0001
Pair No. 3						
Group (intervention/control)	0.9512	(0.61–1.48)	−0.050	0.2262	−0.221	0.824
Nationality	1.2738	(0.55–2.90)	0.242	0.4200	0.576	0.564
Sex	0.6803	(0.45–1.02)	−0.385	0.2069	−1.862	0.062
Pair No. 4						
Group (intervention/control)	1.349	(0.88–2,06)	0.299	0.217	1.376	0.168
Nationality	0.513	(0.22–1.14)	−0.667	0.411	−1.623	0.104
Sex	1.399	(0.94–2.07)	0.336	0.199	1.683	0.092
